# Examining Educational and Career Transition Points Among a Diverse, Virtual Mentoring Network

**DOI:** 10.1007/s41979-024-00119-y

**Published:** 2024-04-04

**Authors:** Erika L. Thompson, Toufeeq Ahmed Syed, Zainab Latif, Katie Stinson, Damaris Javier, Gabrielle Saleh, Jamboor K. Vishwanatha

**Affiliations:** 1 School of Public Health, University of North Texas Health Science Center, 3500 Camp Bowie Blvd, Fort Worth, TX 76107, USA; 2 Institute for Health Disparities, University of North Texas Health Science Center, Fort Worth, TX 76107, USA; 3 McWilliams School of Biomedical Informatics, University of Texas Health Science Center at Houston, Houston, TX 77030, USA; 4 McGovern Medical School, University of Texas Health Science Center at Houston, Houston, TX 77030, USA

**Keywords:** Mentoring, Social capital, Transitions, Higher education, Diversity

## Abstract

Given the differences in trajectory for under-represented minorities in biomedical careers, we sought to explore how a virtual mentoring program, the National Research Mentoring Network (NRMN), and its platform (MyNRMN), may facilitate transitions in the science, technology, engineering, mathematics, and medicine (STEMM) pipeline. The purpose of this study was to describe how the size of an MyNRMN member’s mentoring network and level of engagement correlate with academic and career transitions. We examined MyNRMN platform user data from March 2020 to May 2021 (*n* = 2993). Logistic regression estimated the odds of a career or academic transition related to NRMN mentoring network size and engagement, while adjusting for confounders. Among active MyNRMN users for the one-year period, 5.2% (*n* = 155) had a positive transition. In the adjusted logistic regression model, users with more engagement on the MyNRMN platform (201 + points) had significantly higher odds of a positive transition (aOR = 2.19, 95% CI 1.14, 4.22) compared to those with 1–50 NRMN Points. Network size was not statistically significant. This study shows an association between being active within a virtual mentoring network and positive educational and career transitions in the STEMM pipeline. Active engagement in a virtual mentoring network may have benefits for mentees who aspire to join the STEMM workforce.

## Introduction

The National Research Mentoring Network (NRMN) is a National Institutes of Health (NIH)-funded initiative that provides individuals across all career stages in the biomedical, behavioral, clinical, and social sciences with evidence-based culturally responsive mentoring, networking, and professional development (Ahmed et al., 2021; Javier et al., 2021; Sorkness et al., 2017). NRMN is part of the larger NIH consortium effort, the Diversity Program Consortium (DPC), with an overarching goal of “developing, implementing, assessing and disseminating innovative, effective approaches to research training and mentoring” (National Institutes of Health, n.d., para. 1). Through these resources of evidence-based, culturally responsive mentoring, networking, and professional development, NRMN aims to support individuals’ academic and career transition from one level to the next.

Through the use of MyNRMN, NRMN’s online platform, which houses the resources exclusive to NRMN’s over 27,900 members (as of October 2023), we are able to provide “a national online mentoring and networking platform that provides online tools to connect NRMN mentors and mentees across the nation” (Ahmed et al., 2021, p. 498). The tools consist of multiple forms of mentoring, such as one-on-one guided virtual mentoring in MyMentor, mentor–mentee, peer, and near-peer mentoring through Find a Mentor, group mentoring in My Groups, My Courses which allows mentees and mentors to enroll and take courses such as the NRMN Unconscious Bias Course, Ask a Mentor which allows mentees to pose a question to the entire MyNRMN community, and a Jobs & Internships board among others (Ahmed et al., 2021).

In addition to the resources, MyNRMN utilizes gamification to encourage and measure engagement within the network. Gamification has been defined as the practice of incorporating certain features of video games, such as design and mechanisms, in non-gaming environments (Deterding et al., 2011). Such aspects have been used in online education, mobile applications, and websites, since the “extrinsic motivation, as seen in the form of rewards or other mechanisms, may be necessary to make people engage in the gamifying process” (Kuo & Chuang, 2016, p. 18). In fact, an experiment designed to test whether integrating gamification on a website would increase user engagement versus a website with no gamification aspects showed that users exhibited higher levels of engagement on a gamification-centered website, instead of a non-gamification website (Hsieh & Yang, 2019). MyNRMN’s point-based system supports engagement on the platform for mentors, mentees, and members. When users complete one of the predetermined desired actions, such as completing their profile or publishing a resume, the individuals receive a certain amount of points, which encourages them to complete additional actions. Mentors and mentees are also able to see each other’s point totals and use that to factor into their decision when reaching out to others to grow their network or request mentoring. Part of the rationale for investing in NRMN and MyNRMN is to enhance the diversity of the science, technology, engineering, mathematics, and medicine (STEMM) field, including students and those in the workforce.

### Lack of Diversity in STEMM Field

While there have been strides in diversifying the STEMM fields, women and individuals who are racially underrepresented are more likely to not persist through their education and career in these fields (Packard, 2016). The individuals that are underrepresented in STEMM are Blacks or African Americans, Hispanic/Latinx, American Indians or Alaska Natives, and Native Hawaiians and other Pacific Islanders, individuals with disabilities, individuals from disadvantaged backgrounds, and women (National Institutes of Health, 2019). According to a report from the PEW Research Center, “Blacks make up 11% of the U.S. workforce overall but represent 9% of STEM workers, while Hispanics comprise 16% of the U.S. workforce but only 7% of all STEM workers”—percentages that are not representative of the number of individuals in these communities in total job positions across all sectors in the USA (Funk & Parker, 2018). When examining individuals in the biomedical research workforce, these communities continue to experience underrepresentation at the faculty and Principal Investigator positions levels (Diggs-Andrews et al., 2021).

The lack of representation of historically underrepresented students continues to appear in academia as demonstrated by a report issued by the National Science Foundation’s National Science Board (2018) that confirms in 2015, of all awarded STEM (i.e., engineering, social sciences, psychology, computer sciences, biological/agriculture sciences, physical sciences, mathematics, and statistics) doctoral degrees, only 9% were obtained by underrepresented minority students (URMs; inclusive of Black, Hispanic, American Indian/Alaskan Native), and of all awarded master’s degrees, URMs obtained around 21% of them (National Science Board, 2018). Although URM undergraduate and graduate students continue to remain underrepresented in the STEMM fields, this representation drops even more when examining URM individuals in junior- or senior-level faculty positions, especially within the member institutions of Association of American Medical Colleges (AAMC) (Meyers et al., 2018). Concurrently, while the number of URM postdoctoral fellows has progressively increased over the years, the same cannot be said for URM individuals in faculty positions (Meyers et al., 2018). Interpreting this finding shows that this *transition point*—one that typically precedes an appointment to a faculty role—is less likely to occur for URMs. Even when URM individuals attain STEMM faculty positions, the number of individuals that transition from a junior faculty position to a full professor drops once more, as Meyers et al. (2018) note that merely 4% of all full professors in basic science fields at AAMC universities are URMs.

Increasing the diversity in STEMM is of utmost importance. Diversifying the STEMM workforce will ensure the United States continues to produce scientific and technological innovation as well as improving the country’s health (National Academy of Sciences, 2011; Valantine & Collins, 2015). Additionally, diversifying the STEMM and biomedical workforce ensures equitable opportunities for individuals who have been historically marginalized in these fields (National Academies of Sciences, Engineering, and Medicine, 2023). Further, having a diversity of people, perspectives, and ideas facilitate innovation that has been attributed to the United States’ ability to continue to produce scientific and technological innovation.

### The Need to Promote STEMM Persistence and Progress Through Positive Transitions

In addition to diversifying the STEMM workforce, it is critical to have individuals persist and progress from one career/academic state to another. URM and women in particular can face daunting challenges for persisting in academia (Mendez et al., 2020). However, the recent 30 years have not shown drastic advances in keeping URM faculty in academia, even with endeavors created by organizations throughout the nation aiming to diversify these fields (Martinez et al., 2018). In turn, when URM are not fully represented in faculty positions, URM students may suffer from a lack of guidance from URM faculty who can help them through their academic and professional careers, which can lead to the students not persisting in their STEMM programs (Martinez et al., 2018).

Research has linked *mentoring* as an important support aspect to increase the persistence and progression of individuals as well as a major positive influence in an individual’s career progression and personal and professional development (Burton et al., 2019; Estrada et al., 2018; Haggard et al., 2011; Mendez et al., 2020; Packard, 2016). For example, mentoring can provide valuable support to the development of science identity and a sense of belonging, especially for URM students in predominantly White institutions (Burton et al., 2019). However, there exist disparities regarding access to mentoring due to its typical in-person nature. Specifically, individuals from underrepresented backgrounds, individuals from minority-serving institutions, or under-resourced institutions have less access to mentoring at their current institutions (Segarra et al., 2021). Moreover, many mentoring programs can be race and/or gender neutral, thus reducing the effectiveness of these programs (Salmon, 2022). At the same time, mentoring has also been connected with cultivating “scientific identity,” as shown by a qualitative study which examined this theory in a sample group of underrepresented populations and discovered that “mentors were an important part of identity verification for students” (Atkins et al., 2020, p. 12). Developing scientific identity is vital for retention in the STEM fields, particularly for underrepresented groups, since the concept involves students self-actualizing themselves as scientists, which, in turn, encourages them to persevere through their academic career, from undergraduate school to graduate school and beyond (Atkins et al., 2020).

As previously described, URM STEMM students face greater obstacles in persisting and progressing through academic and career *transition points*, resulting in a loss of diversity in the STEMM workforce and academia. Mentoring can be a strategy to promote persistence through the development of science identity and a sense of belonging; however, not all students have access to mentors within their own institutions, which may require access to virtual mentoring. It is unclear if mentoring in a virtual space has the ability to support these transition points for URM STEMM students. Notwithstanding, the literature has established benefits to mentoring that is offered in a virtual modality. Mentoring offered in a virtual modality has the potential benefit of making STEMM mentoring more accessible and flexible. For example, because of the virtual capabilities, individuals are not limited by the resources and availability of mentors at their institution or physical location (Walsh, 2015). To this end, since individuals are not bound by locations, even if a mentor or mentee relocates, the relationship can continue, facilitating long-term relationships (Speer et al., 2021). Additionally, there is a flexibility component, as individuals do not need to travel and can schedule their interactions at their convenience (Walsh, 2015). Moreover, the recent COVID-19 pandemic showcased the importance of offering services, such as mentoring, in a virtual modality to enhance continued support and flexibility despite external disruptions (Bouchey et al., 2021).

### Research Question and Hypotheses

Given NRMN’s extensive large national mentoring network and portfolio of resources, it is an ideal program to understand career progression and diversification of the STEMM workforce, and we are able to explore how a mentoring platform may facilitate transitions in the STEMM pipeline. Herein lies an opportunity to assess if and how MyNRMN’s resources, including mentorship, are benefiting the STEMM community. We seek to answer the following research question: how is the size of an MyNRMN member’s mentoring network and level of engagement on the platform correlated with academic and career transitions? Our *hypotheses* are that larger MyNRMN member’s mentoring network sizes and higher levels of engagement will demonstrate positive career and/or academic transitions.

## Methods

### Sample

MyNRMN was formed as part of the National Research Mentoring Network as a resource to support online mentoring connections and professional development that could expand opportunities and resources for underrepresented groups in biomedical sciences (Ahmed et al., 2021). NRMN participants are recruited through multiple modalities, including conferences, organizations, universities, and other means of social marketing. The sample for this paper included MyNRMN users who logged in between March 3, 2020, and May 26, 2021. This date range was selected because a new feature was created on the platform to allow users to update their profiles starting in March 2020. The sample included 10,714 users who had the opportunity to have a career, education, or degree transition during this time period. The dataset was subset to those active users who have had some level of engagement on the platform, *n* = 2993. All data for this study were de-identified. Analysis was approved by the North Texas Regional Institutional Review Board.

### Measures

We collected transition points data through our profile data forms. In MyNRMN, we have a rich profile that includes fields such as first name, last name, role, email, alternate email, cellphone, current institution, city, state, zip code, citizenship, education level, career level, degrees, major/field of study, parent/legal guardian attended college, gender, race, ethnicity, sexual identity, disability, which organization referred you, how did you first hear about NRMN, and about me. When the user creates their account, during the enrollment process, they fill out these profile fields or they can always go back to their profile page on the MyNRMN platform under their settings to edit any of the fields. In March 2020, we started capturing the history of changes to this profile page. We also started capturing fields that are related to the career or degree changes (i.e., role, institution, alternate email, city, state, zip code, education level, career level, degrees, major/field of study). When any of these fields changed, we marked that change, timestamped it, and kept both the older field and the newer field change. A positive transition point was designated by a change in one of the following fields: education, degree, career, and role. If a user had a change in any one of these categories, they were labeled as having a positive transition overall. Table 1 describes the types of transitions by field. A transition from a mentee to mentor role was included as a positive transition as this recognizes a shift in development of a person’s training or career to mentor others. Note that all data collected were passively collected as part of the MyNRMN platform rather than active data collection for the purposes of research. The benefit of this form of data capture is it permits ongoing evaluation of the MyNRMN platform; however, it can also result in missing data.

Additional variables of interest for this analysis included gender (male; female; other, prefer not to report, or missing), race (White; Black or African American; Asian; other, prefer not to answer, or missing), ethnicity (non-Hispanic; Hispanic; prefer not to answer or missing), and parent or legal guardian attended college (yes or no). On the MyNRMN platform, we built in a feature called NRMN Points to capture the engagement of the members on the platform. Just like how gamification is employed by other online platforms and video games (Gentry et al., 2019), users earn points based on actions they take on the platform; some actions earn them more points than others. Table 2 below describes in detail the different actions a user can take and the point value they can earn for each of those actions. The idea of NRMN Points is to quantify the engagement of a member, mentor, or mentee, and use this metric to create a better search and recommendation for users. NRMN Points quantify if a member is more engaged or less engaged with our platform (Table 2). NRMN Points were heuristically allocated after reviewing popular online platforms and video game gamification models, and considering the user effort level required for these tasks and value these tasks provide to other users of the network, for example, a user completing their NRMN profile will receive more points than a simple task of clicking on a “like” button. As gamification has shown to increase engagement on online platforms and video games, the initial feedback we have heard from our community is that they like the idea of earning more points, and it encourages them to be more engaged with the platform and to revisit the platform often. For this manuscript, we are using the NRMN Points to gauge and understand the engagement of the participant while they are using the MyNRMN platform. For example, NRMN Points can indicate a person’s use of MyNRMN online resources (e.g., building a CV, MyMentor Program). Given the distribution of the data (median = 50, interquartile range = 50–150, skewness = 7.94, kurtosis = 114.8), NRMN Points were categorized as follows: 1–50 NRMN Points, 51–200 NRMN Points, and 201 + NRMN Points. We also measured network size for each user. This was the number of other users the user was connected to on the platform. Having connections from different backgrounds and with varying skill sets can advance the member’s career opportunities (Ahmed et al., 2021). There are multiple ways members can connect with each other on MyNRMN, like using the robust search engine or the system’s recommendations to browse members’ profiles and connect with members who share interests. Also, mentees can connect to Mentors in MyMentor, a guided virtual mentoring program, and mentors and mentees can use the Find A Mentor feature to connect with mentors or peer mentors (Ahmed et al., 2021). Based on the distribution of the network size data (median = 0, interquartile range = 0–1, skewness = 16.57, kurtosis = 445.17), network size was operationalized as follows: 0 connections, 1–3 connections, and more than 3 connections.

### Data Analysis

Data analysis was performed in SAS version 9.4. Univariate descriptive statistics were estimated for all variables of interest. Bivariate comparisons were performed by the transition outcome variable. Three types of logistic regression models were estimated for this study, and each produced odds ratios and 95% confidence intervals for odds of a positive transition. First, to estimate the independent effect of each of the covariates, unadjusted logistic regression models were created for all covariates. Next, we estimated regression models for model building. Given that mentor network size and engagement (i.e., NRMN Points) were hypothesized as key variables for having a positive transition, each covariate was added to a model with mentor network size and engagement as predictor variables to determine if these affected the main measures of effect. The final adjusted model included mentor network size, NRMN Points, ethnicity, race, gender, role, career, and education.

## Results

Among active MyNRMN users for the 1-year period, 5.2% had a positive transition. The most common transition was a change in mentoring role followed by education, career, and degree (Table 3). Given that users are not required to enter all demographic information for a profile, some users may have missing data for demographic characteristics. Within the sample, 39% identified as female and 17% as male, 40% identified as non-Hispanic and 10% as Hispanic, and 44% identified as White, followed by 23% Black/African American, 15% Asian, and 10% Other. Note that the remaining proportions for each variable reflect missing data. Users had a range of educational levels and career types entered. Approximately four out of ten users were mentors.

To answer the study hypothesis, we estimated the unadjusted logistic regression models for mentor network size and NRMN Points, respectively. Users with more connections were significantly more likely to have a positive transition compared to users without any connections (1–3 connections OR = 3.06, 95%CI 2.05, 4.57; more than 3 connections OR = 6.01, 95%CI 3.89, 9.27). Users with more NRMN engagement were also significantly more likely than users with the lowest levels of engagement to have a positive transition (51–200 NRMN Points OR = 3.76, 95%CI 2.48, 5.72; 201 + NRMN Points OR = 7.28, 95%CI 4.65, 11.39; reference group 1–50 NRMN Points).

In the adjusted logistic regression model, users with more engagement on the NRMN platform with 201 + points had significantly higher odds of a positive transition compared to those with 1–50 NRMN Points (Table 4). However, mentor network size was not statistically significant when controlling for covariates. Users that had higher odds of a positive transition were Black/African American users compared to White users; Hispanic users compared to non-Hispanic users; postdocs, graduate students, and other education levels compared to undergraduates; and students and postdocs compared to faculty. Given that users are not required to enter some fields of information, persons with missing gender data were less likely to have a positive transition compared to males.

## Discussion

This study aimed to explore how engagement and connections from a national program that facilitates a virtual mentoring and networking platform supported career and educational transitions among users. Since the initial launching of the profile update feature on MyNRMN on March 3, 2020, 5% of active users have had a positive educational or career transition. We had hypothesized that increased engagement and larger virtual mentoring networks would be significantly associated with a positive transition. Based on this analysis and adjusting for confounders, active NRMN participants, through the MyNRMN platform, engagement was significantly related to positive transitions.

In regard to platform engagement, websites have been known to incorporate a point-based gamification system to track their level of engagement with their users; concurrently, it can also promote continued engagement due to the external desire it offers to the users through obtaining these points (Kuo & Chuang, 2016). Consequently, MyNRMN uses gamification to encourage and measure engagement within the network. While users may not have made any connections to other mentors or mentees on MyNRMN to have a broad network, they can still engage with the platform via file sharing, discussions, and sharing profile information. Within the network, the most engaged users were the most likely to have a positive transition. Based on these findings, adding in additional gamification components that specifically connect to enhancing positive transitions through professional development and mentoring for the highest level of career stages (e.g., postdoc to faculty) may be useful for the platform. Moreover, there is a need to specifically test the effectiveness of online engagement for mentoring compared to in-person interactions, thus requiring prospective studies on this topic (Gernert et al., 2020).

Given that almost half of the positive transitions observed during this time period were a change from mentee to mentor, MyNRMN users may be benefiting from online resources to develop personal mentoring skills and mentoring networks. This finding speaks to the underlying mentoring life cycle framework for a MyNRMN user. The goal is to create a space for mentees to connect with mentors and receive mentoring via discussion or messaging. Importantly, mentees can select from identities and experiences to support their STEMM goals and path, which moves beyond gender/race neutral mentoring programs (Salmon, 2022). A prior virtual program found that having a mentor matched on gender or race was important to students, and mentees reported receiving more assistance from mentors when matched based on these identities (Blake-Beard et al., 2011). Subsequently, users can then transition into a mentor role to continue the cycle and give back to future biomedical trainee generations. This mentoring life cycle is beneficial for future mentoring programs to consider as it can promote sustainability and reduce the burden on mentors over time. Future studies can examine the specific type of engagement (e.g., use of a program or resource) that contributes to positive transitions.

Moreover, our results demonstrate that positive transitions were more likely to occur among historically underrepresented minority groups, such as Black or African American users and Hispanic users on the MyNRMN platform. The impetus for developing the MyNRMN platform was to facilitate mentoring connections for historically underrepresented groups given that diverse networks may not be available at every local institution and promote equity for transition occurrence (Meyers et al., 2018). Other mentoring programs have relied on virtual technology to expand engagement of mentoring relationships beyond a singular institution and support the removal of geographic barriers (Coates et al., 2004; Keeler et al., 2018; Miller et al., 2008; Valentin-Welch. 2016). Moreover, virtual platforms are not necessarily a hindrance to creating social support and forming relationships (Petersen et al., 2020). Thus, MyNRMN facilitates a paradigm shift from localized mentoring to a national, virtual network of mentors, facilitating accessible mentoring to individuals who may otherwise not have access to mentoring. Future research should explore how the diversity of a user’s mentoring network may impact career and education transitions as hallmarks of successful mentoring.

This study also identified an interesting paradox. When examining the education level of the MyNRMN user, persons who identified as postdoc were significantly more likely to have a positive transition compared to undergraduates; however, when compared to persons in the career field, postdocs were significantly less likely to have a positive career transition compared to faculty. Previous research has demonstrated that although an increase in underrepresented minority PhDs has occurred, the rate of growth is not on par with the number of underrepresented minority Assistant Professors in basic sciences (Gibbs et al., 2016). Postdoctoral fellowships may be an opportunity to increase transitions to assistant professor positions, and NIH-funded programs such as MOSAIC (Maximizing Opportunities for Scientific and Academic Independent Careers) and FIRST (Faculty Institutional Recruitment for Sustainable Transformation) may facilitate this process. In relation, the literature highlighted the need for more diverse individuals at the faculty and Principal Investigator levels in STEMM research (Diggs-Andrews et al., 2021). Herein lies an opportunity to further examine how best NRMN can support individuals at the postbaccalaureate level to facilitate a positive career transition. Some examples of potential support to provide to individuals transitioning from post-baccalaureate include training, professional development, and intentional peer mentoring with more established post-baccalaureates or early career faculty.

These findings should be considered within the context of the data limitations. First, MyNRMN users were not required to enter all demographic information when completing profiles. As a result, there is a substantial amount of missing data for gender, race, ethnicity, career, and educational data. We elected not to conduct a complete case analysis as this would bias the results. Moreover, there may not have been enough time during the study period for a user to have a positive transition and data were from personal profile fields updated by the user. The data also did not permit us to examine unsuccessful attempts at a transition, nor did we consider transitions outside of concrete academic or career milestones. Future analyses should explore the impact of MyNRMN over longer periods of time when additional transition data are available. Finally, while MyNRMN engagement and transitions were significantly associated, the authors cannot rule out the possibility of reverse causality. In other words, people who are more engaged on the platform may be more likely to update their information with relevant transition information.

## Conclusions

Overall, this study shows an association between being active within a virtual mentoring network and positive educational and career transitions in the STEMM pipeline. These findings are of importance to the field for two primary reasons. First, it demonstrates NRMN as a platform that can be leveraged by individuals, colleges, universities, organizations, and professional societies to provide virtual mentoring and support to mentees at all education levels with the goal of increasing the diversity of the STEMM workforce. Additionally, it describes an innovative approach for supporting the diversity of the STEMM training and workforce population that could be implemented in other countries and expanded to additional disciplines.

## Figures and Tables

**Table 1:** Transition types

Type of change	Categories
Career	Assistant—Associate Professor Associate—Full Professor Instructor—Assistant Professor or Researcher Post Doc—Assistant Professor, Faculty, Instructor, Scientist, or Workforce Workforce—PostDoc or Assistant Professor
Education	Bachelors—Masters, Doctoral, or Workforce PostBac—Doctoral Masters—Doctoral Doctoral—Workforce or PostDoc PostDoc—Workforce Workforce—Masters, Doctoral, or PostDoc
Degree	Associates—Bachelors Bachelors—Masters or Doctoral Masters—Doctoral Terminal + (already had a terminal degree, and received another graduate degree)
Role	Mentee—Mentor

**Table 2:** MyNRMN point system

Action	Description	Point value
Comment like	User gets a “like” for a comment	0.5
Comment reply	User gets a “reply” to a comment	1.25
Like	User “likes” a comment	2
Flag	User “flags” a comment	2
User’s group comment	Received for every comment posted to the user’s group	2.5
File	User shares a “file” in a comment	5
Reply	User “replies” to a comment	5
Comment	User posts a comment	10
Star	User “stars” a comment	10
Invite	User invites another user to connect or participate in a group on NRMN	10
User connection	A user’s request to connect is confirmed OR a user confirms a connection	10
Mentor connection	A user’s request to “Request Mentoring” is confirmed OR a Mentor confirms a connection	50
MyMentor connection	A mentees mentoring request is confirmed or a Mentor confirms a connection in MyMentor	50
MyMentor	MyMentor completed	100
Profile complete	User has completed personal profile	50
Published resume	A user has created and published a personal resume	100
Onboarding complete	User has completed all onboarding steps	200

**Table 3:** Frequencies of NRMN transitions

Transition type	*N* (%)
Education	54 (1.8)
Career	51 (1.7)
Degree	23 (0.8)
Role	72 (2.4)
Number of transitions	
0	2838 (94.8)
1	121 (4.0)
2	25 (0.8)
3	7 (0.2)
4	2 (0.1)

**Table 4:** Odds of transition among users for an online mentoring platform (*N* = 2993)

	Total *N* (%)	No transition *N* (%)	Transition *N* (%)	Adjusted OR (95% CI)
		2838 (94.8)	155 (5.2)	
Network size				
0	1668 (55.7)	1628 (57.4)	40 (25.8)	Reference
1–3	944 (31.5)	878 (30.9)	66 (42.6)	1.40 (0.84, 2.33)
More than 3	381 (12.7)	332 (11.7)	49 (31.6)	1.40 (0.74, 2.65)
NRMN Points				
1–50	1656 (55.3)	1622 (57.2)	34 (21.9)	Reference
51–200	944 (31.5)	875 (30.8)	69 (44.5)	1.61 (0.95, 2.72)
201 +	393 (13.1)	341 (12.0)	52 (33.6)	**2.19 (1.14, 4.22)**
Gender				
Male	512 (17.1)	471 (16.6)	41 (26.5)	Reference
Female	1173 (39.2)	1063 (37.5)	110 (71.0)	1.49 (1.00, 2.21)
Other, missing	1308 (43.7)	1304 (46.0)	4 (2.6)	**0.17 (0.03, 0.88)**
Race				
White	1308 (43.7)	1247 (43.9)	61 (39.4)	Reference
Black/African American	682 (22.8)	639 (22.5)	43 (27.7)	**1.91 (1.21, 3.01)**
Asian	459 (15.3)	443 (15.6)	16 (10.3)	1.04 (0.56, 1.91)
Other	302 (10.1)	285 (10.0)	17 (11.0)	1.08 (0.58, 2.04)
Missing	242 (8.1)	224 (7.9)	18 (11.6)	1.85 (0.96, 3.59)
Ethnicity				
Non-Hispanic	1208 (40.4)	1107 (39.0)	101 (65.2)	Reference
Hispanic	297 (9.9)	258 (9.1)	39 (25.2)	**1.78 (1.08, 2.95)**
Missing	1488 (49.7)	1473 (51.9)	15 (9.7)	0.80 (0.40, 1.61)
Role				
Mentee	1759 (58.8)	1693 (59.7)	66 (42.6)	Reference
Mentor	1234 (41.2)	1145 (40.4)	89 (57.4)	1.40 (0.91, 2.17)
Education				
Undergraduate or Postbac	327 (10.9)	318 (11.2)	9 (5.8)	Reference
Graduate	291 (9.7)	261 (9.2)	30 (19.4)	**4.25 (1.90, 9.52)**
Postdoc	239 (8.0)	206 (7.3)	33 (21.3)	**14.21 (5.17, 39.03)**
Other (currently working/faculty/not in school or formal program)	810 (27.1)	732 (25.8)	78 (50.3)	**4.33 (1.73, 10.80)**
Missing	1326 (44.3)	1321 (46.6)	5 (3.2)	2.03 (0.38, 10.85)
Career				
Faculty	713 (23.8)	637 (22.5)	76 (49.0)	Reference
Staff/other	306 (10.2)	285 (10.0)	21 (13.6)	0.88 (0.50, 1.56)
Postdoc	183 (6.1)	167 (5.9)	16 (10.3)	**0.37 (0.17, 0.80)**
Student	59 (2.0)	48 (1.7)	11 (7.1)	**3.62 (1.43, 9.17)**
Scientist	82 (2.7)	71 (2.5)	11 (7.1)	1.28 (0.60, 2.71)

Bold values indicate statistical significance

## Data Availability

De-identified data may be available upon reasonable request to the authors.
